# Effects of virtual reality exposure on psychological distress in adolescent oncology patients

**DOI:** 10.1177/03008916251356848

**Published:** 2025-10-21

**Authors:** Andrea Paulis, Valerio Marri, Andrea De Salvo, Valentina Di Ruscio, Stefania Gaspari, Maria Debora De Pasquale, Giada Del Baldo, Roberto Premuselli, Michela Origlia, Angela Mastronuzzi, Giuseppe Maria Milano

**Affiliations:** 1 Onco-Hematology, Cell and Gene Therapy, Bambino Gesù Children’s Hospital, IRCCS, Rome, Lazio, Italy; 2 Davide Ciavattini Onlus, Rome, Italy

**Keywords:** Adolescents, young adults, cancer, virtual reality, artificial intelligence

## Abstract

Adolescents and young adults (AYA) with cancer often experience significant psychological distress during hospitalization. Virtual reality (VR) is a non-pharmacological intervention, yet its application in pediatric oncology remains limited. In this study, 35 patients aged 12–21 were assigned to either an experimental group (n=20), which received four VR sessions over two weeks in addition to standard care, or a control group (n=15), which received standard care only. Psychological measures included the Distress Thermometer (DT), State-Trait Anxiety Inventory-Y1 (STAI-Y1), and Hospital Anxiety and Depression Scale (HADS). The experimental group showed significant reductions in distress (DT), state anxiety (STAI-Y1), and depressive symptoms (HADS-D) (all p<.05; Cohen’s d medium to large size). No significant changes were observed in the control group. These findings suggest that VR is a feasible, well-tolerated, and potentially effective tool for reducing psychological distress in AYA oncology patients. Such preliminary results support its integration into multidisciplinary care. Further large-scale studies are needed to confirm its efficacy.

## Introduction

In recent decades, the scientific community has recognized that the Adolescent and Young Adult (AYA) population presents unique epidemiological, clinical, and psychosocial characteristics, to which traditional healthcare services often struggle to respond.^1,2^ AYA patients often have lower access to clinical trials, delayed diagnoses, and frequently face a range of illness-related stressors, including the risk of mortality, loss of physical integrity, the invasiveness of treatments, treatment side effects, the risk of relapse, and the negative impact on psychological and physical functioning.^3^ Consequently, the clinical management of AYA patients with oncological diagnoses represents a significant challenge, which requires a treatment approach that integrates the biopsychosocial aspects of the illness. In this context, the multidisciplinary team plays a crucial role in promoting quality of life during hospitalization, addressing not only medical needs but also the psychological and social needs of patients.^4^

In recent years, specific intervention programs dedicated to AYA patients have been developed, including in Italy by the Italian Association for Pediatric Hematology-Oncology (AIEOP), a national cooperative group which has promoted various targeted interventions such as expressive and creative interventions.^5^

Virtual reality (VR) has emerged as a promising tool in healthcare, offering potential benefits in reducing pain and anxiety among hospitalized patients.^6,7^ However, despite the increasing body of literature supporting the use of VR in clinical settings, research specifically targeting the oncological AYA population, particularly during hospitalization, remains limited.

This study aims to investigate the effects of VR exposure (head-mounted displays) on anxious and depressive symptoms in hospitalized AYA oncology patients. Participants were recruited from the Pediatric Haematology and Oncology Unit, Cell and Gene Therapy of the Bambino Gesù Children’s Hospital, in Rome. Through exposure to immersive and relaxing virtual environments, the study seeks to evaluate changes in anxious and depressive symptoms after four sessions, as well as variations in state anxiety and emotional distress levels before and after each exposure.

It is hypothesized that the use of VR will lead to a reduction in distress, state and non-state anxiety symptoms, and depressive symptoms, as measured through specific psychometric tools. Specifically, we expect: a decrease in state anxiety and emotional distress scores immediately following VR exposure; and a decrease in anxiety and depression scores after the two-week period during which the four VR exposure sessions will take place. Furthermore, we expect to observe significant differences between the two groups of patients, with a significantly greater reduction in the mentioned symptoms in the experimental group.

## Methods

Inclusion criteria were: age between 12 and 21 years; confirmed oncological diagnosis; hospitalization period of at least two weeks; willingness to participate in the study; and provision of signed informed consent. Exclusion criteria were: limited comprehension of the Italian language; visual impairments; restricted neck mobility; medical conditions that could be exacerbated by exposure to digital images (e.g., photosensitive epilepsy or central nervous system tumors); previous diagnosis of psychiatric disorders, documented intellectual disabilities, or neurodevelopmental disorders; and sensory or motor impairments that could prevent the safe use of the virtual reality device.

The study involved a total of 35 subjects (Table 1) between 12 and 21 years (M=15.69; SD=2.23), 17 males (48.6%) and 18 females (51.4%), undergoing oncological treatments at the Paediatric Haematology and Oncology, Cell and Gene Therapy Unit of the Bambino Gesù Children’s Hospital, IRCCS, in Rome, between January and December 2024 (Online Supplementary Table 1, Sample characteristics; Online Supplementary Table 2, Sample’s descriptive statistics).

**Table 1.** Diagnostic and demographic distribution by group.

Diagnosis	Group	n	Male (n)	Female (n)
Acute Myeloid Leukemia (AML)	Experimental	7	4	3
	Control	5	2	3
Acute Lymphoblastic Leukemia (ALL)	Experimental	4	1	3
	Control	6	3	3
Hodgkin Lymphoma (HL)	Experimental	4	2	2
	Control	0	–	–
Non-Hodgkin Lymphoma (NHL)	Experimental	2	1	1
	Control	0	–	–
Primary Mediastinal Large B-Cell Lymphoma (PMBCL)	Experimental	1	1	0
	Control	1	0	1
Refractory Cytopenia of Childhood (RCC)	Experimental	1	0	1
	Control	0	–	–
Burkitt Lymphoma (BL)	Experimental	1	1	0
	Control	0	–	–
Sickle Cell Disease (SCD)	Experimental	1	0	1
	Control	0	–	–
Osteosarcoma	Experimental	1	0	1
	Control	0	–	–
Ewing Sarcoma	Experimental	1	1	0
	Control	3	1	2
Anaplastic Large Cell Lymphoma, ALK-negative (ALK– ALCL)	Experimental	0	–	–
	Control	0	–	–
Total	Experimental	20	11	9
	Control	15	6	9

Patients were divided into an experimental group (N=20) or a control group (N=15) by researchers (Online Supplementary Table 3, Diagnostic and demographic distribution by group).

### Intervention

For the VR exposures used in this study, the Meta Quest 2 headset (Meta Platforms) was used. The sessions, which involved the experimental group after completing the pre-session self-assessment questionnaires, lasted a total of 30 minutes each and were conducted in the patient’s hospital room. During each VR session, participants engaged in three activities that they could choose from a list of pre-selected environments curated by clinicians from Nature Treks VR and Liminal VR applications.

#### Nature Treks VR

Nature Treks VR is a digital app, developed by Greener Games (https://www.greenergames.net/nature-treks), which allows users to explore different scenarios such as tropical beaches, snowy mountains, etc. Within them there is the possibility of controlling weather variables and interacting with the surrounding virtual environment.

Patients enrolled in the experimental group could choose from the following scenarios: “Blue Ocean,” a setting characterized by sandy beaches and calm seas; “Blue Deep,” which offers an immersive experience where users can explore ocean depths; “White Winter,” a snowy mountain environment characterized by towering peaks and snow; “Green Meadows,” a large green valley characterized by flower-filled meadows and lush flora.

#### Liminal VR

**Figure 1.**
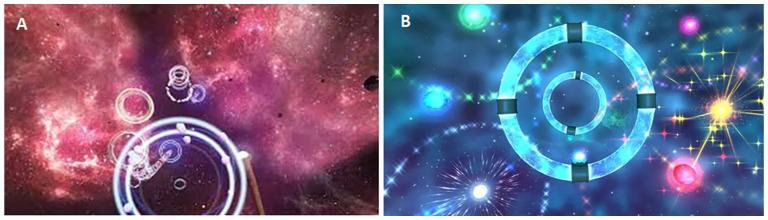
Illustration of the Liminal VR games used. (A) Meridian, (B) Orbit.

Liminal VR, developed by Liminal VR Pty Ltd (https://liminalvr.com/), is an application that immerses users in interactive environments. Patients enrolled in the experimental group could choose between the following games: “Meridian” (Figure 1A) and “Orbit” (Figure 1B). In Meridian, the user wields a wand that, when directed toward moving luminous circles, produces relaxing melodies, while Orbit requires participants to focus on maintaining the alignment between two rings to trigger light effects during sustained alignment.

### Psychological measures

#### Hospital Anxiety and Depression Scale

The Hospital Anxiety and Depression Scale (HADS) is a 14-item self-report questionnaire, validated in Italian, used to assess anxiety and depressive symptoms over the past week of hospitalization.^8,9^ It provides separate scores for anxiety and depression, reflecting the severity of each symptom domain. This questionnaire was administered at baseline, one and two weeks later.

#### Distress Thermometer

The Distress Thermometer (DT) is a tool that allows patients to evaluate their level of emotional distress by selecting a number from 0 (absence of emotional distress) to 10 (maximum emotional distress) on a visual thermometer.^10^ This tool was administered before and after each virtual reality session to measure the impact of VR sessions on distress levels.

#### State-Trait Anxiety Inventory-Y

The State-Trait Anxiety Inventory-Y (STAI-Y) is composed of two subscales^11^: Form Y1, assessing state anxiety, and Form Y2, assessing trait anxiety. In this study, only the Y1 module was used to avoid overburdening patients with psychometric tools. It consists of 20 items rated on a 4-point scale (from 1 = “not at all” to 4 = “very much so”), evaluating anxiety symptoms at the time of completion. The questionnaire was administered before and after each VR session.

### Data analysis

All statistical analyses were performed using SPSS version 27.0. To summarize demographic data descriptive statistics, including means and standard deviations, were calculated.

To compare the mean scores of DT, STAI-Y1, the Depression subscale of the HADS (HADS-D), and the Anxiety subscale of the HADS (HADS-A) between participants in each group, independent samples t-tests were used. Paired samples t-tests were applied to assess the effectiveness of the interventions within each group. Additionally, Cohen’s d was calculated to evaluate the effect size. Results were considered statistically significant when the p-value was ⩽ 0.05.

## Results

### Experimental group

Considering only the subjects in the experimental group, paired t-tests revealed significant reductions in DT and STAI-Y1 scores at all timepoints (T0–T1 to T6–T7), all p < .001, with large effect sizes (Cohen’s d ranging from 1.01 to 1.68). HADS-D score showed significant decreases from T0 to T3 (p = .017; d = 0.584) and from T0 to T7 (p = .015; d = 0.599), both with medium effect sizes. Similarly, HADS-A score significantly decreased from T0 to T3 (p = .012; d = 0.620) and from T0 to T7 (p = .015; d = 0.704), also with medium effect sizes. The detailed results are presented in supplementary materials (Online Supplementary Table 4, Experimental Group paired samples, measured pre- and post-intervention; Online Supplementary Table 5, Effect sizes for the experimental group from pre- to post-intervention).

### Control group

Considering only the subjects in the control group, no significant differences were observed between the measurements for the DT scores, the STAI-Y1, the HADS-D, and the HADS-A. The detailed results are presented in supplementary materials (Online Supplementary Table 6, Control Group paired samples, measured pre- and post-intervention).

### Comparisons between groups

No statistically significant differences were found between the two groups in terms of gender, age, and other characteristics (Online Supplementary Table 7, Gender distribution by group; Online Supplementary Table 8, Chi-square test for gender and group association; Online Supplementary Table 9, Age at Enrolment descriptive stats by group; Online Supplementary Table 10, ANOVA summary for Age at Enrolment; Online Supplementary Table 11, ANOVA results for Age at Enrolment group comparison).

Independent samples t-tests revealed significant between-group differences in DT and STAI-Y1 scores at T1, T3, T5, and T7 (all p < .01), with the experimental group consistently reporting lower scores. No significant differences were found at T0, T2, T4, or T6.

HADS-D scores showed significant differences between groups were observed at T3 (p = .013) and T7 (p = .012), with lower scores recorded in the experimental group. No differences emerged at T0. HADS-A scores did not significantly differ between groups at any time point.

The detailed results are presented in supplementary materials (Online Supplementary Table 12, Independent samples t-test comparing the Experimental and Control groups).

### Limitations

Regarding the limitations of the study, it should be noted that it was a single-centre study, which may limit the broader applicability of the results. Another potential limitation of this study is the small sample size (n = 35). Future research with larger cohorts is necessary to confirm these findings. Longer follow-up periods would help determine whether the observed effects are sustainable over time. Finally, although in the present study patients were given the option to choose between pre-selected VR environments, further studies could explore which types of environments are most effective for specific patient groups. A limitation to the generalizability of the study is that it did not consider gender/sex issues.

## Discussion

The results of this study preliminarily demonstrate the impact of VR, delivered through visors, in reducing symptoms of anxiety, depression, and perceived stress among the population of AYA cancer patients. The comparison made between the experimental group and the control group highlights the usefulness of VR as a potential complementary intervention for the management of psychological distress.

The measurements for DT and STAI-Y1 revealed statistically significant reductions, both within the same group before and after the session, and between the two groups. A possible explanation for these results can be attributed to the distraction effect produced by VR environments.^12^ These environments might promote the absorption of attentional resources, thereby reducing the salience of unpleasant internal stimuli, such as negative thoughts or pain. Variables such as state anxiety are conceived as transitory emotions characterized by physiological arousal, feelings of apprehension and thus susceptible to fluctuations in intensity depending on situational variables.^13,14^ Moreover, studies by Riva et al.^15^ and Annerstedt et al.^16^ showed that virtual natural environments could induce a relaxation response, even at the autonomic level (e.g., reduction in heart rate and muscle tension) in individuals exposed to them.

The comparison between pre and post session scores highlighted statistically significant reductions in scores related to anxiety and depression symptoms within the experimental group. When comparing the experimental and control groups, the decrease in HADS-D scores in the experimental group suggests a significant reduction in depressive symptoms, which was not observed for the HADS-A scores. This discrepancy could be explained by the impact that immersive and interactive activities had on active engagement, including physical involvement in exploring virtual environments, among patients in prolonged hospitalization. The characteristics of VR described in this study could, therefore, have represented a form of Behavioral Activation (BA), a widely used and documented intervention in the treatment of depressive symptoms.^17^

The results of our study underscore the potential of VR as a non-invasive, scalable, and cost-effective intervention for managing psychological distress in adolescent oncology patients. From a cognitive-behavioral perspective, VR could serve as an intervention capable of eliciting BA, thus representing a potential resource for patients with high levels of depressive symptoms.

VR interventions may contribute to an increased sense of confort and subjective control, which are often lacking in these patients. The active engagement required by VR can also facilitate a therapeutic approach. Although the results are promising, it is desirable that the limitations mentioned be addressed in future studies .

## Supplemental Material

sj-pdf-1-tmj-10.1177_03008916251356848 – Supplemental material for Effects of virtual reality exposure on psychological distress in adolescent oncology patients Supplemental material, sj-pdf-1-tmj-10.1177_03008916251356848 for Effects of virtual reality exposure on psychological distress in adolescent oncology patients by Andrea Paulis, Valerio Marri, Andrea De Salvo, Valentina Di Ruscio, Stefania Gaspari, Maria Debora De Pasquale, Giada Del Baldo, Roberto Premuselli, Michela Origlia, Angela Mastronuzzi and Giuseppe Maria Milano in Tumori Journal
