# Supplementary material for: Effects of virtual reality exposure on psychological distress in adolescent oncology patients
Source: Tumori. 2025 Oct 21;111(6):529–34. doi: 10.1177/03008916251356848 (PMC12638458; doi:10.1177/03008916251356848)
Supplement: sj-pdf-1-tmj-10.1177_03008916251356848 – Supplemental material for Effects of virtual reality exposure on psychological distress in adolescent oncology patients [file sj-pdf-1-tmj-10.1177_03008916251356848.pdf]

“Effects of Virtual Reality Exposure on Psychological Distress in Adolescent Oncology Patients: A Pilot Study”
SUPPLEMENTARY MATERIAL

Group

		Frequency	Percentage	Valid Percentage	Cumulative Percentage
Valid	Experimental	20	57,1	57,1	57,1
	Control	15	42,9	42,9	100,0
	Total	35	100,0	100,0	

Gender

		Frequency	Percentage	Valid Percentage	Cumulative Percentage
Valid	M	17	48,6	48,6	48,6
	F	18	51,4	51,4	100,0
	Total	35	100,0	100,0	

Descriptive Statistics

	N	Min	Max	Mean	Standard Deviation
Age at Enrollment	35	12	21	15,69	2,233
Valid (listwise)	35				

Contingency Table * Group

			Group		Total
			Experimental	Control	
Gender	M	Count	11	6	17
		% within Gender	64,7%	35,3%	100,0%
		Stand. residual	,4	-,5	
	F	Count	9	9	18
		% within Gender	50,0%	50,0%	100,0%
		Stand. residual	-,4	,5	
Total		Count	20	15	35
		% within gender	57,1%	42,9%	100,0%

Chi-Square Test

	Value	df	Asymptotic Sig. (2 way)	Exact Sig (2 ways)	Exact Sig (1 way)
Pearson Chi-Square	,772[a]	1	,380		
Continuity Correction [b]	,288	1	,591		
Likelihood Ratio	,776	1	,378		
Fisher's Exact Test				,500	,296
Linear-by-Linear Association	,750	1	,386		
Number of valid cases	35				

Report

Age at Enrollment

Group	Mean	N	Standard Dev.
Experimenta l	16,10	20	2,100
Control	15,13	15	2,356
Total	15,69	35	2,233

ANOVA

			Sum of Squares	df	Mean Square
Age at Enrollment * Group	Between	(Combined)	8,010	1	8,010
	Within		161,533	33	4,895
	Total		169,543	34	

ANOVA

			F	Sig.
Age at Enrollment * Group	Between	(Combined)	1,636	,210
	Within			
	Total			

Group Statistics

	Group	N	Mean	Standard dev	t	Sig.
DistressT0	Experimental	20	4,30	2,408	-,474	,639
	Control	15	4,67	2,059		
DistressT1	Experimental	20	2,80	1,852	-2,942	,006

	Control	15	4,73	2,017		
DistressT2	Experimental	20	3,80	2,397	-,742	,463
	Control	15	4,40	2,324		
DistressT3	Experimental	20	2,15	1,663	-3,326	,002
	Experimental	15	4,33	2,225		
DistressT4	Control	20	2,60	1,818	-1,858	,072
	Experimental	15	3,93	2,434		
DistressT5	Control	20	1,35	1,348	-3,569	,002
	Experimental	15	3,93	2,549		
DistressT6	Control	20	3,45	2,564	-,792	,434
	Experimental	15	4,13	2,475		
DistressT7	Control	20	1,75	2,099	-3,122	,004
	Experimental	15	4,20	2,541		

Group Statistics

	Group	N	Mean	Standard dev	t	Sig.
STAI_T0	Experimental	20	35,95	11,185	-1,150	,259
	Control	15	40,67	13,048		
STAI_T1	Experimental	20	27,40	5,195	-3,817	,001
	Control	15	40,60	12,614		
STAI_T2	Experimental	20	33,20	6,646	-1,472	,150
	Control	15	37,47	10,474		
STAI_T3	Experimental	20	27,40	4,773	-3,476	,003
	Control	15	37,07	9,946		
STAI_T4	Experimental	20	34,25	8,058	-1,112	,277
	Control	15	38,13	11,581		
STAI_T5	Experimental	20	26,70	5,332	-3,749	,001

	Control	15	38,53	11,319		
STAI_T6	Experimental	20	33,55	8,581	-,949	,349
	Control	15	36,67	10,854		
STAI_T7	Experimental	20	26,70	7,954	-3,114	,004
	Control	15	36,40	10,494		

Group Statistics

	Group	N	Mean	Standard dev.	t	Sig.
HADS_T0	Experimental	20	5,55	4,419	-1,176	,248
	Control	15	7,33	4,467		
HADS_T3	Experimental	20	3,95	3,034	-2,613	,013
	Control	15	7,27	4,480		
HADS_T7	Experimental	20	3,45	2,328	-2,773	,012
	Control	15	7,13	4,734		
Anxiety_T0	Experimental	20	6,20	3,764	,188	,852
	Control	15	5,93	4,605		
Anxiety_T3	Experimental	20	4,05	2,544	-1,214	,234
	Control	15	5,33	3,716		
Anxiety_T7	Experimental	20	3,80	3,254	-1,020	,315
	Control	15	5,07	4,096		

Paired Samples Statistics

		Mean	N	Standard dev.	t	
Pair 1	DistressT0	4,30	20	2,408	5,430	,000
	DistressT1	2,80	20	1,852		

Pair 2	DistressT2	3,80	20	2,397	5,320	,000
	DistressT3	2,15	20	1,663		
Pair 3	DistressT4	2,60	20	1,818	5,784	,000
	DistressT5	1,35	20	1,348		
Pair 4	DistressT6	3,45	20	2,564	6,240	,000
	DistressT7	1,75	20	2,099		
Pair 5	STAI_T0	35,95	20	11,185	4,515	,000
	STAI_T1	27,40	20	5,195		
Pair 6	STAI_T2	33,20	20	6,646	6,472	,000
	STAI_T3	27,40	20	4,773		
Pair 7	STAI_T4	34,25	20	8,058	7,501	,000
	STAI_T5	26,70	20	5,332		
Pair 8	STAI_T6	33,55	20	8,581	6,333	,000
	STAI_T7	26,70	20	7,954		
Pair 9	HADS_T0	5,55	20	4,419	2,610	,017
	HADS_T3	3,95	20	3,034		
Pair 10	HADS_T0	5,55	20	4,419	2,677	,015
	HADS_T7	3,45	20	2,328		
Pair 11	HADS_T3	3,95	20	3,034	1,291	,212
	HADS_T7	3,45	20	2,328		
Pair 12	Anxiety_T0	6,20	20	3,764	2,772	,012
	Anxiety_T3	4,05	20	2,544		
Pair 13	Anxiety_T0	6,20	20	3,764	3,148	,005
	Anxiety_T7	3,80	20	3,254		

Pair 14	Anxiety_T 3	4,05	20	2,544	,592	,561
	Anxiety_T 7	3,80	20	3,254		

Paired Samples Statistics

		Mean	N	Standard dev	t	SIg.
Pair 1	DistressT0	4,67	15	2,059	-1,000	,334
	DistressT1	4,73	15	2,017		
Pair 2	DistressT2	4,40	15	2,324	1,000	,334
	DistressT3	4,33	15	2,225		
Pair 3	DistressT4	3,93	15	2,434	,000	1,000
	DistressT5	3,93	15	2,549		
Pair 4	DistressT6	4,13	15	2,475	-1,000	,334
	DistressT7	4,20	15	2,541		
Pair 5	STAI_T0	40,67	15	13,048	,193	,849
	STAI_T1	40,60	15	12,614		
Pair 6	STAI_T2	37,47	15	10,474	,634	,536
	STAI_T3	37,07	15	9,946		
Pair 7	STAI_T4	38,13	15	11,581	-1,572	,138
	STAI_T5	38,53	15	11,319		
Pair 8	STAI_T6	36,67	15	10,854	1,293	,217
	STAI_T7	36,40	15	10,494		
Pair 9	HADS_T0	7,33	15	4,467	,155	,879
	HADS_T3	7,27	15	4,480		
Pair 10	HADS_T0	7,33	15	4,467	,248	,808
	HADS_T7	7,13	15	4,734		

Pair 11	HADS_T3	7,27	15	4,480	,269	,792
	HADS_T7	7,13	15	4,734		
Pair 12	Anxiety_T0	5,93	15	4,605	,963	,352
	Anxiety_T3	5,33	15	3,716		
Pair 13	Anxiety_T0	5,93	15	4,605	,982	,343
	Anxiety_T7	5,07	15	4,096		
Pair 14	Anxiety_T3	5,33	15	3,716	,552	,589
	Anxiety_T7	5,07	15	4,096		

Effect Size (Pre-post experimental group)

STAI_T0	STAI_T1	Cohen's d	1.009
STAI_T2	STAI_T3	Cohen's d	1.447
STAI_T4	STAI_T5	Cohen's d	1.677
STAI_T6	STAI_T7	Cohen's d	1.416
DistressT0	DistressT1	Cohen's d	1.214
DistressT2	DistressT3	Cohen's d	1.190
DistressT4	DistressT5	Cohen's d	1.293
DistressT6	DistressT7	Cohen's d	1.395
HADS_T0	HADS_T3	Cohen's d	0.584
HADS_T0	HADS_T7	Cohen's d	0.599
HADS_T3	HADS_T7	Cohen's d	0.289
Anxiety_T0	Anxiety_T3	Cohen's d	0.620
Anxiety_T0	Anxiety_T7	Cohen's d	0.704
Anxiety_T3	Anxiety_T7	Cohen's d	0.132

Effect Size : experimental group vs control group

DistressT0	Cohen’s d	-0.1618
DistressT1	Cohen’s d	-1.0050
DistressT2	Cohen’s d	-0.2535
DistressT3	Cohen’s d	-1.1361
DistressT4	Cohen’s d	-0.6345
DistressT5	Cohen’s d	-1.3248
DistressT6	Cohen’s d	-0.2704
DistressT7	Cohen’s d	-1.0665
STAI_T0	Cohen’s d	-0.3927
STAI_T1	Cohen’s d	-1.4485
STAI_T2	Cohen’s d	-0.5029
STAI_T3	Cohen’s d	-1.3025
STAI_T4	Cohen’s d	-0.3999
STAI_T5	Cohen’s d	-1.4071
STAI_T6	Cohen’s d	-0.3243
STAI_T7	Cohen’s d	-1.0638
HADS_T0	Cohen’s d	-0.4017
HADS_T3	Cohen’s d	-0.8923
HADS_T7	Cohen’s d	-1.0366
Anxiety_T0	Cohen’s d	0.0644
Anxiety_T3	Cohen’s d	-0.4145
Anxiety_T7	Cohen’s d	-0.3484
